# Disproportionate reporting of euglycaemic and diabetic ketoacidosis with SGLT-2 inhibitors across expanding cardiorenal indications: a cross-database study of FAERS and JADER

**DOI:** 10.3389/fendo.2026.1957734

**Published:** 2026-09-03

**Authors:** Jinqian Chen, Deyin Wang, Xiaochuan Duan, Jianbo Wang, Zhenyu Zhao, Xiaolong Xing

**Affiliations:** 1 NHC Key Laboratory of Hormones and Development, and Tianjin Key Laboratory of Metabolic Diseases, Chu Hsien-I Memorial Hospital & Tianjin Institute of Endocrinology, Tianjin Medical University, Tianjin, China; 2 Tianjin Medical College, Tianjin, China; 3 School of Medicine, Nankai University, Tianjin, China

**Keywords:** disproportionality analysis, euglycaemic diabetic ketoacidosis, FAERS, JADER, pharmacovigilance, SGLT-2 inhibitors

## Abstract

SGLT-2 inhibitors are now used beyond type 2 diabetes (T2DM) in heart failure (HF) and chronic kidney disease (CKD), where glucose is monitored less routinely. Whether their established ketoacidosis signal—particularly euglycaemic diabetic ketoacidosis (euDKA)—is maintained as the indications expand, and whether it reproduces across independent reporting systems, is untested. We analysed the US FAERS (2020Q1–2026Q1) and Japanese JADER using one pipeline. Signals required consensus across four methods (ROR, PRR, IC, EBGM/EB05). Analyses were run overall, within report-level indication strata [T2DM, HF, CKD, off-label type 1 diabetes (T1DM)] and against an active comparator (DPP-4 inhibitors), with two pre-specified negative controls, time-to-onset modelling and sensitivity analyses. The DKA signal met four-method consensus within every indication stratum, including HF and CKD, and was highest in off-label T1DM; it was therefore maintained, not diluted, as indications expanded. Overall RORs were 67.4 (95% CI 65.7–69.2) in FAERS and 112.3 (104.8–120.3) in JADER. SGLT-2 inhibitors accounted for 85.8% (FAERS) and 91.7% (JADER) of all euDKA reports, though euDKA was only 4.1% and 7.8% of SGLT-2 inhibitor reports. Both negative controls were null in both databases. Median time-to-onset was 60 days (Weibull β=0.48, 95% CI 0.46–0.50). Across two independent reporting systems, the SGLT-2 inhibitor ketoacidosis signal was maintained across the expanding cardiorenal indications, with euDKA concentrated within this class and early onset. Ketone-based assessment is warranted when ketoacidosis is suspected, particularly soon after initiation. As a disproportionality analysis, these are hypothesis-generating signals that cannot establish incidence, relative risk, or causality.

## 1 Introduction

Sodium-glucose co-transporter-2 (SGLT-2) inhibitors were introduced for glycaemic control in type 2 diabetes but have since been approved for heart failure and chronic kidney disease (1–3), where their benefits extend to patients across a range of metabolic backgrounds. As prescribing expands into these cardiorenal indications, SGLT-2 inhibitors are increasingly used in clinical settings in which routine glucose monitoring is less central than in diabetes care, and in which the index of suspicion for a metabolic emergency may be lower. Understanding how the safety profile behaves as the indications broaden has therefore become clinically important.

Diabetic ketoacidosis (DKA) is a recognised adverse effect of SGLT-2 inhibitors (4, 5). Of particular concern is euglycaemic DKA (euDKA), in which ketoacidosis develops while blood glucose is only mildly elevated or near-normal; because the usual hyperglycaemic alarm is absent, euDKA is prone to delayed recognition and can be precipitated by intercurrent illness, fasting, surgery or volume depletion; although uncommon in absolute terms (well under one per thousand SGLT-2 inhibitor users in randomised and observational cohorts), its life-threatening nature and diagnostic difficulty make it a priority safety concern (6–9). Because the underlying mechanism is a drug-level metabolic effect, the risk may not be confined to diabetes.

Spontaneous-reporting databases such as the US FDA Adverse Event Reporting System (FAERS) and the Japanese Adverse Drug Event Report database (JADER) are well suited to characterising such signals, but gaps remain. First, although euDKA has been examined against other glucose-lowering agents (10), the magnitude of the euDKA association relative to the entire reporting background, and its reproducibility in an independent reporting system, remain unquantified. Second, most pharmacovigilance analyses have aggregated reports across indications (10, 11); the one analysis that did separate indications compared two agents across two groups (diabetes versus heart failure) within a single national reporting system (12), leaving unresolved whether the DKA signal is diluted or maintained across the full range of cardiorenal indications, where diabetes is common but not universal. Third, although a median onset of approximately one month has been reported (10), the shape of the onset-time distribution has not been characterised at scale, limiting the ability to define a high-risk surveillance window after initiation.

To address these gaps, we conducted an indication-stratified disproportionality study of SGLT-2 inhibitor–associated DKA and euDKA in FAERS, with JADER as an independent replication. We separated euDKA from DKA, stratified by indication (type 2 diabetes, heart failure, chronic kidney disease and off-label type 1 diabetes), used DPP-4 inhibitors as an active comparator and two pre-specified negative controls (fracture and road traffic accident), and examined time-to-onset with sensitivity analyses for suspect-drug role, acute precipitants and calendar period. This distinguishes the present work from database-wide analyses that ranked drugs associated with ketoacidosis without isolating the euglycaemic phenotype, stratifying by indication, or replicating in a second system (13).

## 2 Materials and methods

This was a hypothesis-generating pharmacovigilance signal-detection study based on spontaneous reports, not an incidence-based epidemiological analysis. The analysis plan was pre-specified before data extraction. Reporting follows the READUS-PV statement for disproportionality analyses (14) and STROBE for observational studies (15); a completed READUS-PV checklist is provided.

### 2.1 Data sources and case selection

This was a disproportionality analysis of individual case safety reports (ICSRs). Two spontaneous-reporting databases were analysed in parallel: the US FDA Adverse Event Reporting System (FAERS, quarterly extracts, 2020Q1–2026Q1; https://fis.fda.gov/extensions/FPD-QDE-FAERS/FPD-QDE-FAERS.html) as the primary source (16), and the Japanese Adverse Drug Event Report database (JADER, cumulative file, May 2026; https://www.pmda.go.jp/safety/info-services/drugs/adr-info/suspected-adr/0004.html) for cross-database replication (17). The FAERS window opens at the first SGLT-2 inhibitor heart-failure approval, encompassing the period in which the cardiorenal indications were approved and adopted while beginning several years after launch to limit the early-marketing (Weber) reporting surge. The FAERS window was closed at 2026Q1 (31 March 2026); a fixed analytic dataset was used, and quarterly updates released after this date were not incorporated. FAERS duplicates were removed by retaining, within each CASEID, the record with the most recent CASEVERSION/PRIMARYID, and deleted-case reports were excluded; in JADER each identifier corresponds to a single case.

### 2.2 Exposure, comparator and indication

Exposure was defined as any SGLT-2 inhibitor recorded as a suspect drug (FAERS role PS/SS; JADER “suspected drug”), including fixed-dose combinations; the full agent list for each database is given in the Supporting Information. Dipeptidyl peptidase-4 (DPP-4) inhibitors recorded as suspect drugs served as the active comparator. Indication strata [type 2 diabetes (T2DM), heart failure (HF), chronic kidney disease (CKD), and off-label type 1 diabetes (T1DM)] were defined at the report level: a report entered a given stratum when that condition appeared among the recorded drug indications. Report-level assignment was used because drug-level indication fields were too sparsely populated to support stable stratified estimation (the chronic kidney disease stratum contained a single comparator event) (FAERS INDI; JADER reason-for-use), allowing a report to contribute to more than one stratum.

### 2.3 Outcomes

The primary outcome was a pre-specified diabetic ketoacidosis (DKA) cluster of MedDRA preferred terms confined to drug-relevant ketoacidosis (diabetic ketoacidosis, euglycaemic diabetic ketoacidosis, ketoacidosis, ketosis and metabolic acidosis; Japanese MedDRA equivalents for JADER) (18). Terms representing non-drug ketoacidosis, other acidoses and isolated laboratory findings were excluded; the full preferred-term list for every cluster is given in the **Supporting Information** (**Supplementary Table 1**). euDKA was defined by the euglycaemic preferred term specifically. Reported euDKA frequencies therefore refer to cases coded with the euglycaemic diabetic ketoacidosis preferred term; euglycaemic events coded only as diabetic ketoacidosis would not be captured, which may underestimate the true number of euglycaemic cases. Two pre-specified negative controls (fracture; road traffic accident) were analysed separately as pipeline checks.

### 2.4 Statistical analysis

Disproportionality was assessed with four complementary methods: the reporting odds ratio (ROR) (19) with 95% confidence interval, the proportional reporting ratio (PRR) (20), the Bayesian information component (IC) with its 2.5% lower bound (IC025), and the multi-item gamma-Poisson shrinker (MGPS) (21), summarised by the empirical Bayes geometric mean (EBGM) and its 5% lower bound (EB05) (22). A robust signal required consensus across all four methods; calculation formulas and decision thresholds for all four methods are given in the **Supporting Information** (**Supplementary Table 6**), with the contingency cell counts underlying every estimate in **Supplementary Table 2**. Time-to-onset was computed only for reports with complete (YYYYMMDD) therapy-start and event dates; reports missing either date, or with non-positive intervals or intervals exceeding 10 years, were excluded. This four-method consensus guards against the method-dependent false-positive signals that any single algorithm can produce (23). All analyses were performed in R version 4.4.2 (R Foundation for Statistical Computing), with openEBGM for the MGPS/EBGM computation.

Analyses were run overall (SGLT-2 inhibitor versus all other drugs), within each report-level indication stratum, and as an active-comparator contrast against DPP-4 inhibitors. To characterise euDKA, two complementary proportions were computed: an exposure-background proportion (euDKA reports among all SGLT-2 inhibitor reports) and a case-attribution proportion (SGLT-2 inhibitor reports among all euDKA reports). Time-to-onset (TTO) from therapy start to event was modelled with a Weibull distribution; a shape parameter β < 1 indicates an early-failure (front-loaded) pattern. Two pre-specified sensitivity analyses were applied to both databases: (i) restriction to reports in which an SGLT-2 inhibitor was the primary suspect drug, and (ii) exclusion of reports listing acute precipitants of ketosis. As a further sensitivity analysis, the DKA cluster was broadened to return the acidosis and ketone-related preferred terms excluded from the primary definition (**Supplementary Table 1**), to test whether the signal depended on the restrictive term selection. A calendar-period analysis split FAERS reports into 2020–2022 and 2023–2026. The same pipeline was applied to JADER and directional concordance assessed across databases. Grammarly (Grammarly Inc.) was used for language editing only; all code, analyses, and interpretations were produced and verified by the authors, who take full responsibility for the scientific content. Generative AI was not used for authorship. all analyses, results and interpretations were verified by the authors, who take full responsibility for the content.

## 3 Results

### 3.1 Overall disproportionality and pipeline specificity

**Figure 1:**
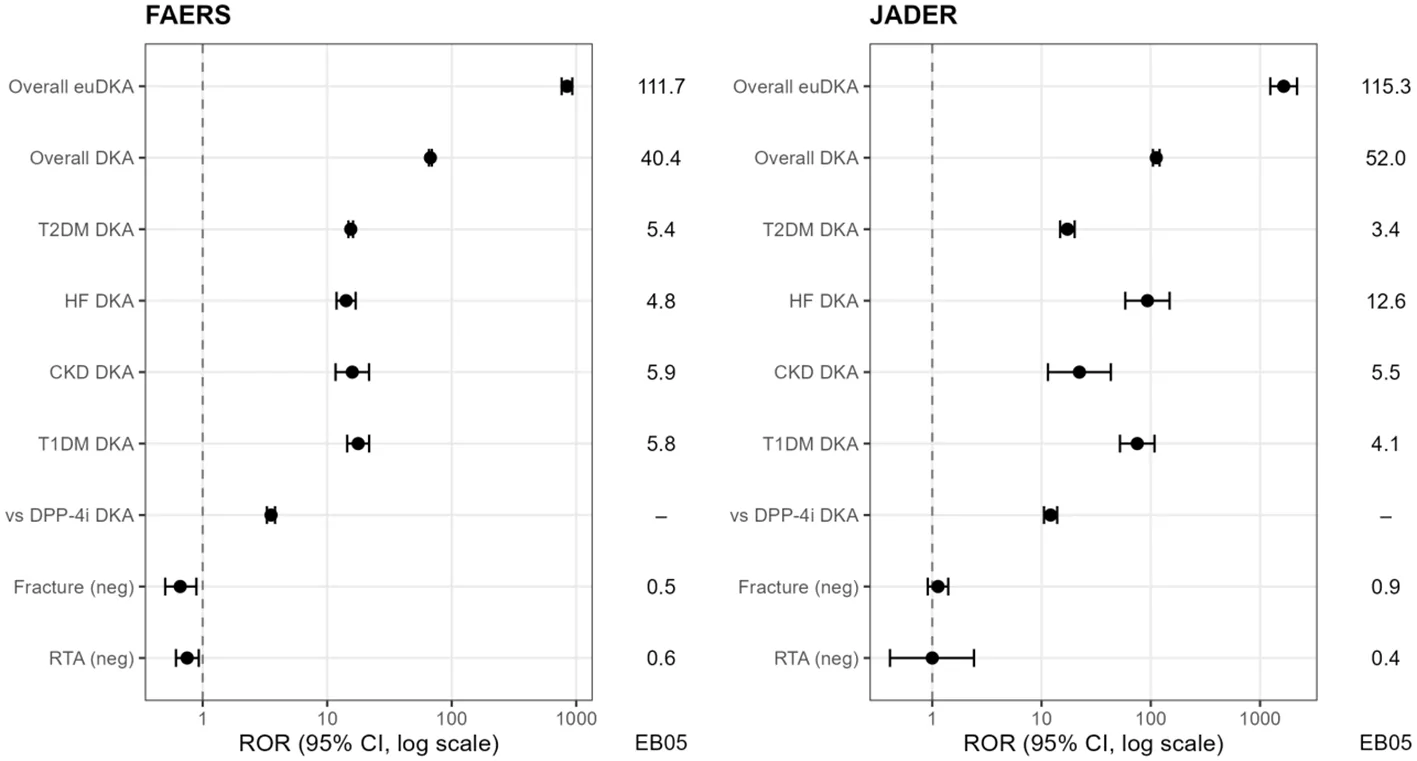
Disproportionality of diabetic and euglycaemic ketoacidosis with SGLT-2 inhibitors across FAERS (left) and JADER (right). Points are reporting odds ratios (ROR) with 95% confidence intervals on a logarithmic scale; the dashed line marks ROR = 1. Rows show the overall DKA and euDKA signals, the indication-stratified DKA signals (T2DM, HF, CKD, off-label T1DM), the active-comparator contrast versus DPP-4 inhibitors, and the two pre-specified negative controls (fracture; road traffic accident). Both negative controls were null in both databases, supporting pipeline specificity.

After de-duplication, approximately 9.1 million reports were analysed in FAERS and 1,032,027 in JADER; SGLT-2 inhibitors were a suspect drug in 67,465 and 7,508 reports, respectively (**Supplementary Figure 1**). The DKA cluster was strongly over-reported with SGLT-2 inhibitors in both databases (FAERS ROR 67.4, 95% CI 65.7–69.2; JADER 112.3, 104.8–120.3), meeting all four criteria (FAERS EBGM 41.1, EB05 40.4; JADER EBGM 54.3, EB05 52.0). The euDKA signal was larger still (FAERS ROR 843, 764–931, EBGM 115.2, EB05 111.7; JADER 1645, 1241–2182, EBGM 123.4, EB05 115.3). Both pre-specified negative controls showed no signal by any method in either database (FAERS: fracture ROR 0.66, 0.50–0.89, EB05 0.51; road traffic accident ROR 0.75, 0.61–0.93, EB05 0.62. JADER: fracture ROR 1.13, 0.91–1.40, EB05 0.91; road traffic accident ROR 1.00, 0.41–2.41, EB05 0.37), supporting pipeline specificity (**Figure 1**; **Table 1**).

**Table 1:** Overall four-method disproportionality.

Database/outcome	N	ROR (95% CI)	PRR	IC025	EBGM	EB05	Consensus
FAERS — DKA	8,779	67.4 (65.7–69.2)	✓	✓	41.1	40.4	4/4
FAERS — euDKA	2,783	843 (764-931)	✓	✓	115.2	111.7	4/4
FAERS — fracture (neg. control)	45	0.66 (0.50–0.89)	NS	NS	0.65	0.51	0/4
FAERS — road traffic accident (neg. control)	89	0.75 (0.61–0.93)	NS	NS	0.74	0.62	0/4
JADER — DKA	1,535	112.3 (104.8–120.3)	✓	✓	54.3	52.0	4/4
JADER — euDKA	589	1645 (1241–2182)	✓	✓	123.4	115.3	4/4
JADER — fracture (neg. control)	85	1.13 (0.91–1.40)	NS	NS	1.09	0.91	0/4
JADER — road traffic accident (neg. control)	5	1.00 (0.41–2.41)	NS	NS	0.75	0.37	0/4

### 3.2 Reporting frequency of euDKA

Two complementary proportions characterised the euDKA association. euDKA represented a small fraction of all SGLT-2 inhibitor reports [FAERS 4.1% (2,783/67,465); JADER 7.8% (589/7,508)], indicating a low reporting proportion (which does not represent an absolute frequency, incidence, or clinical probability, given the absence of a population denominator). Conversely, among all euDKA reports in each database, SGLT-2 inhibitors accounted for the large majority [FAERS 85.8% (2,783/3,244); JADER 91.7% (589/642)], indicating that euDKA reporting was concentrated overwhelmingly within this drug class (**Table 2**).

**Table 2:** Reporting frequency of euglycaemic DKA with SGLT-2 inhibitors.

Database	euDKA among all SGLT-2i reports (background)	SGLT-2i among all euDKA reports (attribution)
FAERS	4.1% (2,783/67,465)	85.8% (2,783/3,244)
JADER	7.8% (589/7,508)	91.7% (589/642)

euDKA, euglycaemic diabetic ketoacidosis. The background proportion reflects the low reporting proportion of euDKA among SGLT-2 inhibitor reports; the attribution proportion reflects the concentration of euDKA reporting within this class.

### 3.3 Indication-stratified analysis

Within every indication stratum, SGLT-2 inhibitors met four-method consensus for DKA in both databases (**Table 3**). In FAERS the magnitude was similar across type 2 diabetes, heart failure and chronic kidney disease and highest in off-label type 1 diabetes; in JADER the same four strata were all positive, with the highest estimates in heart failure and off-label type 1 diabetes. The signal was therefore maintained, rather than diluted, as the recorded indication moved from diabetes to cardiorenal disease.

**Table 3:** Indication-stratified DKA, four methods.

Database/indication	N	ROR (95% CI)	PRR	IC025	EBGM	EB05	Consensus
FAERS — T2DM	5,733	15.5 (14.8–16.1)	✓	✓	5.53	5.41	4/4
FAERS — HF	360	14.2 (11.9–16.9)	✓	✓	5.28	4.84	4/4
FAERS — CKD	95	15.9 (11.7–21.7)	✓	✓	7.04	5.93	4/4
FAERS — off-label T1DM	252	17.7 (14.5–21.7)	✓	✓	6.47	5.83	4/4
JADER — T2DM	875	17.3 (14.8–20.1)	✓	✓	3.59	3.40	4/4
JADER — HF	103	93.2 (58.5–148.6)	✓	✓	14.89	12.63	4/4
JADER — CKD	25	22.2 (11.5–43.1)	✓	✓	7.80	5.54	4/4
JADER — off-label T1DM	253	75.2 (52.3–108.1)	✓	✓	4.59	4.14	4/4

ROR, reporting odds ratio; PRR, proportional reporting ratio; IC025, lower 2.5% bound of the information component; EBGM, empirical Bayes geometric mean; EB05, lower 5% bound of EBGM. ✓ indicates the method’s signal criterion was met. EBGM/EB05 from openEBGM.

### 3.4 Active-comparator analysis

Relative to DPP-4 inhibitors, SGLT-2 inhibitors were disproportionately associated with DKA in both databases (FAERS ROR 3.54, 95% CI 3.28–3.81; JADER 12.1, 10.5–13.9; four-method consensus met; **Supplementary Table 3**), indicating that the signal is not explained by the diabetes-reporting context shared by glucose-lowering drugs.

### 3.5 Time to onset

**Figure 2:**
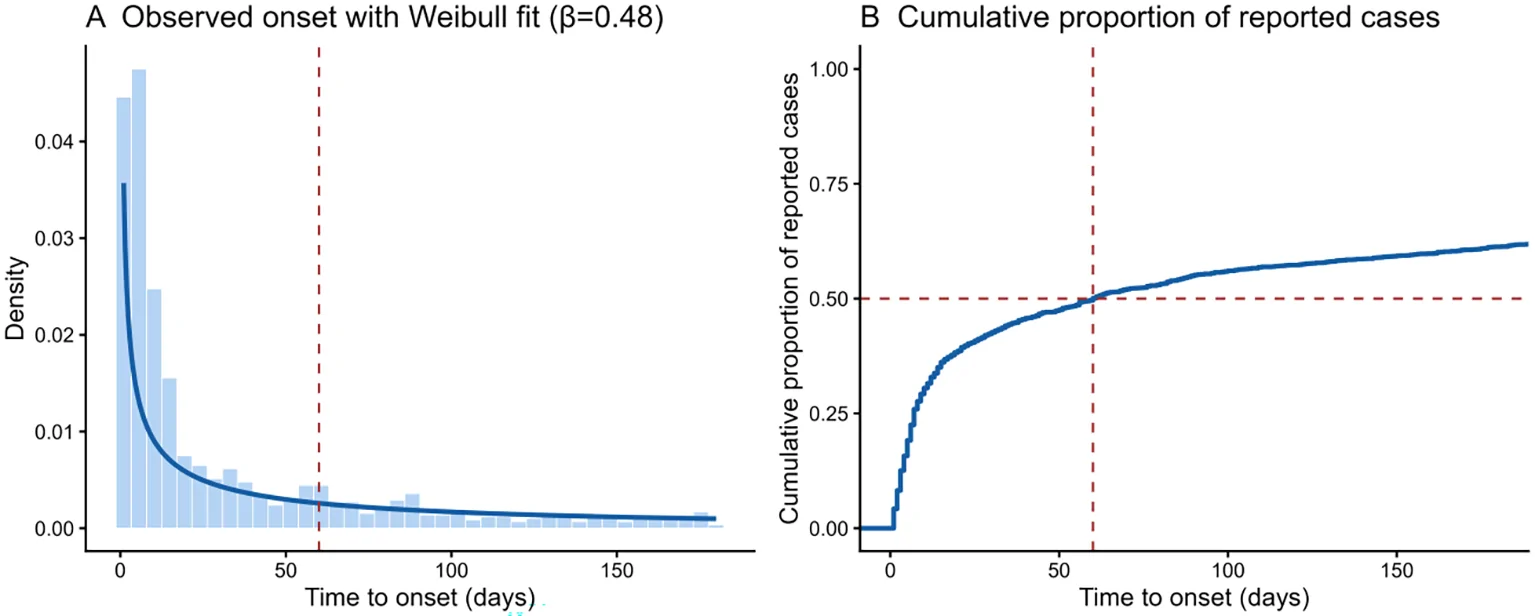
Time to onset of diabetic ketoacidosis after SGLT-2 inhibitor initiation (FAERS; n=2,080). **(A)** Observed onset-time distribution (bars) with the fitted Weibull density (line); β=0.48 (95% CI 0.46–0.50) indicates an early-failure pattern. **(B)** Empirical cumulative proportion of reported cases. The dashed line marks the median onset (60 days); the x-axis is truncated at 180 days to display the early window (interquartile range 7–449 days).

Among 2,080 of the 8,779 FAERS DKA reports with evaluable timing (23.7%), the median interval from SGLT-2 inhibitor initiation to DKA was 60 days (interquartile range 7–449); the Weibull shape parameter was β=0.48 (95% CI 0.46–0.50; upper bound <1), indicating an early-failure pattern in which reported onset was more frequently observed earlier after initiation among cases with available timing data (**Figure 2**).

### 3.6 Sensitivity analyses

The signals were robust to two pre-specified sensitivity analyses applied to both databases. Restricting to reports in which an SGLT-2 inhibitor was the primary suspect drug attenuated magnitudes modestly but preserved a significant DKA signal in every stratum. Excluding reports listing acute precipitants did not reduce the signal and, in the HF stratum, increased it (FAERS HF ROR 14.2→20.1; overall 67.4→80.1), arguing against acute illness or starvation ketosis as the principal explanation (**Supplementary Table 5**). The signal also persisted when the outcome definition was broadened to return the excluded acidosis and ketone-related terms to the DKA cluster: the overall ROR under the wide definition was 40.5 (95% CI 39.6–41.5) in FAERS and 56.9 (53.5–60.5) in JADER, versus 67.4 (65.7–69.2) and 112.3 (104.8–120.3) under the primary definition, with all four methods still met in both databases (**Supplementary Table 9**). The lower magnitude under the wide definition reflects the inclusion of non-specific acidoses (alcoholic, starvation and lactic) that are not preferentially reported with SGLT-2 inhibitors, indicating that restriction to the tightened cluster increased specificity without generating the signal.

### 3.7 Calendar-period analysis

Because the FAERS window opens six to seven years after launch, the early-marketing surge is largely avoided. Split into 2020–2022 and 2023–2026, the within-indication DKA signal remained significant in both periods and in every stratum (overall ROR 123.7 then 41.2; HF 23.7 then 8.4; CKD 16.1 then 16.7); point estimates were higher in the earlier period, as expected for a newer exposure, but the persistence of a strong signal in the most recent period argues against post-approval reporting growth (the Weber effect) as the explanation (**Supplementary Table 4**).

### 3.8 Cross-database concordance

**Figure 3:**
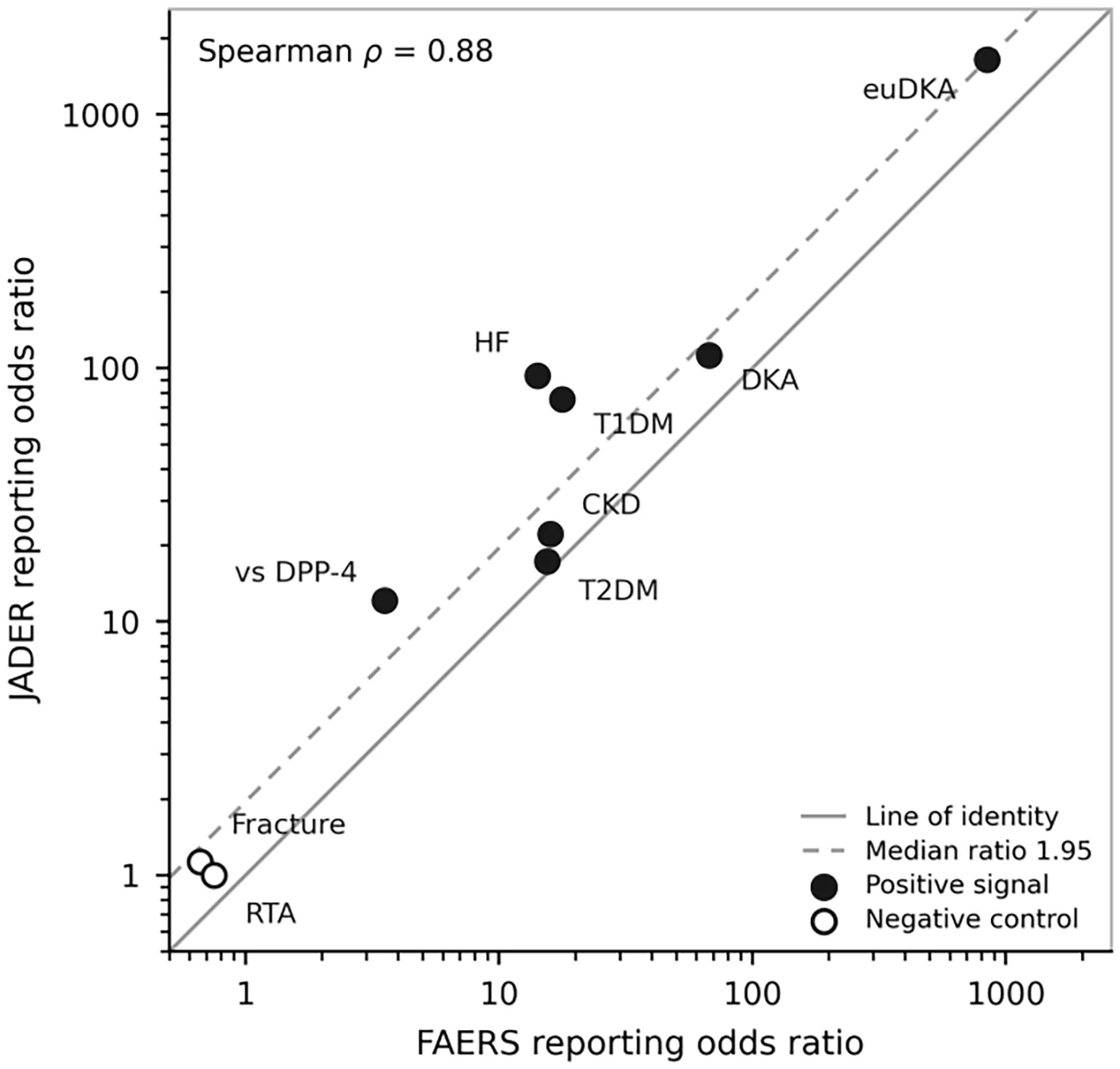
Comparison of reporting odds ratios between FAERS and JADER for the nine pre-specified paired comparisons. Filled circles denote positive signals; open circles denote the two pre-specified negative controls. Both axes are on a logarithmic scale. The dashed line is the line of identity; the dotted line marks the median JADER-to-FAERS ratio of 1.95. JADER estimates were systematically higher, with the largest ratios in the strata containing the fewest reports, while the rank ordering was preserved.

Across the pre-specified comparisons—overall DKA and euDKA, four indication strata, the DPP-4 inhibitor contrast and two negative controls—FAERS and JADER were directionally concordant for every positive signal and both returned null negative controls (**Figure 1**). Across the nine paired comparisons, JADER estimates were systematically higher than FAERS (median ratio 1.95; range 1.12–6.57), although the magnitude of disproportionality estimates is not directly comparable across databases; the rank ordering was nonetheless preserved (Spearman ρ=0.88, p=0.002; **Figure 3**; **Supplementary Table 8**). The largest ratios occurred in the two strata with the fewest reports (heart failure 6.57; off-label type 1 diabetes 4.24), whereas the best-populated comparisons diverged least (type 2 diabetes 1.12; overall DKA 1.67). Cross-database agreement is therefore interpreted at the level of direction and signal status rather than effect size.

## 4 Discussion

In two large, independent spontaneous-reporting systems, ketoacidosis was a consistent and prominent signal for SGLT-2 inhibitors. Three findings stand out. First, euglycaemic DKA was reported overwhelmingly with this drug class, an association strong enough to behave as a near-defining feature of the event. Second, the DKA signal was not confined to type 2 diabetes but was maintained, with four-method consensus, across heart failure, chronic kidney disease and off-label type 1 diabetes. Third, onset was concentrated soon after initiation.

These signals were directionally concordant across two systems that differ in drug-use patterns, populations and regulatory environments, which reduces the likelihood that the association is an artefact of one database. It does not, however, substitute for denominator-based study: both systems lack a true exposed population and are susceptible to variable reporting completeness and to notoriety and indication-related reporting, so our estimates quantify disproportionality of reporting, not incidence or causal risk.

It is important to distinguish the contrast used here from an indication-to-indication contrast. Within each stratum we compared SGLT-2 inhibitors with all other drugs, asking whether the class stands out for DKA in that clinical context—a different question from whether DKA is reported more often in one indication than another. Both can hold simultaneously.

The indication-stratified results require careful interpretation. Database indications reflect the recorded context of use, not the metabolic state of individual patients, so our findings show that the DKA signal is maintained when SGLT-2 inhibitors are reported in cardiorenal contexts; they cannot establish risk specifically in non-diabetic individuals. The practical implication stands: as prescribing expands into settings where glucose is monitored less routinely, the ketoacidosis signal does not diminish.

Several analyses argue against the most plausible confounders. The signal persisted when analysis was restricted to reports in which an SGLT-2 inhibitor was the primary suspect drug, and excluding reports listing acute precipitants did not attenuate it. It also remained significant across calendar periods, and our analytic window, opening years after launch, limits the influence of the early-marketing (Weber) reporting surge. The consistently null negative controls further support the specificity of the analytic pipeline. The elevated signal in off-label type 1 diabetes, a setting in which the class is not approved and for which dedicated risk-management guidance exists (24), likely reflects both a true drug effect and the underlying ketosis-prone physiology of these patients, which the present design cannot disentangle.

### 4.1 Mechanistic interpretation

The pattern is coherent with the established pharmacology of SGLT-2 inhibition: blocking SLC5A2 induces persistent glucosuria, and the resulting fall in plasma glucose lowers insulin and raises the glucagon-to-insulin ratio (7, 25). Crucially, because glucose continues to be excreted, ketoacidosis can develop while blood glucose remains only mildly elevated—the defining feature of euDKA. This pattern is biologically plausible given the established pharmacology and is consistent with a drug-level metabolic predisposition rather than a disease-specific effect, supported by two observations: euDKA reporting was concentrated within this class, and the DKA signal was maintained at a broadly similar level across all four indication strata. The two euDKA proportions carry distinct clinical meaning: the exposure-background proportion (euDKA among all SGLT-2 inhibitor reports; 4.1% in FAERS, 7.8% in JADER) reflects how frequently euDKA appears among adverse-event reports for the class, whereas the case-attribution proportion (SGLT-2 inhibitors among all euDKA reports; 85.8% and 91.7%) reflects the near-exclusive contribution of this class to euDKA reporting, and neither represents an incidence. The very high euglycaemic-DKA reporting odds ratio follows from this near-class-specificity—euDKA is rarely reported for other drugs, so even a modest number of reports yields a large disproportionality estimate—and quantifies reporting disproportionality rather than a multiplicative increase in individual risk.

The same framework accounts for the temporal and sensitivity findings: onset clusters soon after initiation, and excluding reports listing acute precipitants did not attenuate the signal, compatible with such factors amplifying rather than causing an underlying drug-driven tendency. We emphasise that, as a disproportionality study, these data cannot demonstrate causation; the mechanistic account and accompanying schematic are a biologically plausible interpretation, not a pathway demonstrated by these data (**Supplementary Figure 2**).

### 4.2 Clinical implications

The early-onset pattern points to the initiation period—and to triggers such as intercurrent illness, fasting and the perioperative setting—as the situations warranting the greatest vigilance. Because euglycaemia removes the usual hyperglycaemic warning, clinicians should not be reassured by a normal or near-normal glucose when a patient on an SGLT-2 inhibitor presents with malaise, nausea or dyspnoea; ketone measurement, rather than glucose alone, should guide assessment. This applies across the expanding cardiorenal indications, not only in diabetes care. Spontaneous-reporting databases predominantly capture clinically recognised events and cannot identify asymptomatic or subclinical ketone elevation. Complementary real-world evidence is therefore informative: a recent cross-sectional study of 211 outpatients with diabetic kidney disease receiving SGLT-2 inhibitors directly measured capillary β-hydroxybutyrate and identified asymptomatic ketonaemia, with heart failure among the independent predictors of ketone positivity (26). This offers a clinical counterpart to the present pharmacovigilance signal, illustrating a spectrum from subclinical ketone elevation—which would not ordinarily generate an adverse-event report—to clinically recognised ketoacidosis.

### 4.3 Comparison with prior work

Two earlier FAERS analyses examined ketoacidosis with SGLT-2 inhibitors directly. Blau et al. provided an early characterisation of the signal shortly after the class was marketed (4), and He et al. subsequently compared euDKA and DKA reporting for SGLT-2 inhibitors against other glucose-lowering agents ^10^. The present study differs in three respects. First, our reference group is the entire reporting background rather than a single therapeutic class, so the disproportionality is not conditioned on the safety profile of the comparator class; a within-class contrast against DPP-4 inhibitors is retained as a secondary analysis. Second, we replicate the analysis in JADER, an independent reporting system, and require concordance across four detection methods including Bayesian shrinkage, with pre-specified negative controls to test pipeline specificity. Third, we stratify by recorded indication to test whether the signal is maintained as prescribing extends into heart failure, chronic kidney disease and off-label type 1 diabetes—an axis earlier analyses, performed when the class was used predominantly in type 2 diabetes, could not address.

Closest to the present analysis in design, Sakamoto et al. compared the adverse-event profiles of empagliflozin and dapagliflozin between reports from patients with diabetes and those with heart failure in JADER, and identified ketoacidosis in both groups (12). That analysis establishes that indication-related differences in the reported safety profile exist. It did not, however, separate the euglycaemic phenotype from classic ketoacidosis, extend beyond two agents and two indication groups, apply detection methods other than the reporting odds ratio, or test whether the pattern reproduces in a second reporting system. The present study addresses each of these points and adds chronic kidney disease and off-label type 1 diabetes as further strata, so that the behaviour of the signal can be followed across the full range of indications into which the class has expanded.

Our findings should be read alongside a recent FAERS analysis by Yeung et al., who compared the descriptive frequency of euglycaemic ketoacidosis between SGLT-2 inhibitor adverse-event reporters with and without heart failure and found no excess in the heart-failure group (27). That analysis asked a different question with a different denominator: within reporters already exposed to an SGLT-2 inhibitor, whether heart failure raised the frequency of euglycaemic ketoacidosis. We instead compare the class against the entire reporting background. A null within-exposed contrast is compatible with strong disproportionality against background, and our heart-failure stratum retained a substantial signal in both databases—indicating that the association is maintained, not created, when the indication shifts to a cardiorenal setting.

Our study is distinct from recent disproportionality analyses of drug-associated ketoacidosis. A comprehensive FAERS analysis by Du et al. characterised ketoacidosis across many drug classes at the level of the whole database (13). We instead isolate a single class, separate euglycaemic from classic DKA, stratify by recorded indication, add an active comparator and negative controls, stress-test the signal (suspect-only, precipitant-excluded and calendar-period analyses), and replicate it in a second, independent database—extending that aggregate work toward a granular, robustness-tested characterisation of euDKA specifically.

Broader class-level analyses have similarly reported ketoacidosis signals across newer glucose-lowering drug groups in FAERS (11). Such analyses establish that ketoacidosis reporting is not confined to a single agent, but do not isolate the euglycaemic presentation, test whether it persists as indications expand, or replicate it independently.

### 4.4 Limitations

This study has limitations beyond those inherent to spontaneous reporting. Indications and event terms are recorded inconsistently, and euDKA may be under-coded where the euglycaemic qualifier is omitted, so our euDKA counts are conservative. The CKD stratum, and the heart-failure stratum in JADER, rest on comparatively few reports with correspondingly wide confidence intervals, and these estimates should be regarded as exploratory. Because indications were assigned at the report level, a report listing more than one qualifying condition contributed to more than one stratum, so the strata are overlapping rather than mutually exclusive and reflect recorded treatment context rather than individual metabolic state. Accordingly, our findings describe persistence of the reporting signal within HF- and CKD-associated reports rather than a defined clinical risk in HF or CKD patient populations. Consistent with this, among SGLT-2 inhibitor reports only 20.6% of HF-associated and 28.2% of CKD-associated reports also listed a diabetes indication in FAERS (23.9% and 24.1%, respectively, in JADER); these strata are therefore predominantly, but not exclusively, use outside recorded diabetes and should not be read as diabetes-free cohorts. Time-to-onset could be evaluated in 2,080 of 8,779 SGLT-2 inhibitor DKA reports (23.7%); reports with usable dates differed modestly from those without (**Supplementary Table 7**), so the onset estimates describe this subset rather than all reports. Disproportionality magnitudes are not comparable across databases, and JADER, as a cumulative file, is not time-aligned with the FAERS window; cross-database comparison is therefore interpreted qualitatively. Finally, because spontaneously reported cases lack a defined denominator, these estimates quantify reporting patterns, not incidence or population risk. The natural next step is a denominator-based, real-world cohort that can estimate the incidence of DKA and laboratory-confirmed euDKA and characterise onset timing across indications; such validation would convert these consistent signals into quantified risk. Recent commentary from the group that developed READUS-PV sets out minimum requirements for a disproportionality study to merit further consideration, including transparency of methods, stability under stress-testing of expected biases, and cautious choice of the reporting period (28); the present analysis was designed against those requirements. Case-level causal assessment, which the same framework treats as complementary intrinsic evidence, is precluded here because case narratives are not routinely accessible in either database.

## 5 Conclusion

Across FAERS and JADER, euglycaemic DKA was reported predominantly with SGLT-2 inhibitors, and the DKA signal persisted across the expanding cardiorenal indications with an early onset. These consistent signals support ketone-based vigilance soon after initiation, pending denominator-based confirmation.

## Data Availability

The original contributions presented in the study are included in the article/**Supplementary Material**. Further inquiries can be directed to the corresponding authors.

## References

[1] Heerspink HJ Stefánsson BV Correa-Rotter R Chertow GM Greene T Hou F-F . Dapagliflozin in patients with chronic kidney disease. N Engl J Med. (2020) 383:1436–46. doi: 10.1056/NEJMoa2024816 32970396

[2] McMurray JJV Solomon SD Inzucchi SE Køber L Kosiborod MN Martinez FA . Dapagliflozin in patients with heart failure and reduced ejection fraction. N Engl J Med. (2019) 381:1995–2008. doi: 10.1056/NEJMoa1911303 31535829

[3] Zinman B Wanner C Lachin JM Fitchett D Bluhmki E Hantel S . Empagliflozin, cardiovascular outcomes, and mortality in type 2 diabetes. N Engl J Med. (2015) 373:2117–28. doi: 10.1056/NEJMoa1504720 26378978

[4] Blau JE Tella SH Taylor SI Rother KI . Ketoacidosis associated with SGLT2 inhibitor treatment: Analysis of FAERS data. Diabetes Metab Res Rev. (2017) 33 (8):e2924. doi: 10.1002/dmrr.2924 28736981 PMC5950709

[5] Fadini GP Bonora BM Avogaro A . SGLT2 inhibitors and diabetic ketoacidosis: data from the FDA Adverse Event Reporting System. Diabetologia. (2017) 60:1385–9. doi: 10.1007/s00125-017-4301-8 28500396

[6] Peters AL Buschur EO Buse JB Cohan P Diner JC Hirsch IB . Euglycemic diabetic ketoacidosis: A potential complication of treatment with sodium-glucose cotransporter 2 inhibition. Diabetes Care. (2015) 38:1687–93. doi: 10.2337/dc15-0843 26078479 PMC4542270

[7] Rosenstock J Ferrannini E . Euglycemic diabetic ketoacidosis: A predictable, detectable, and preventable safety concern with SGLT2 inhibitors. Diabetes Care. (2015) 38:1638–42. doi: 10.2337/dc15-1380 26294774

[8] Colacci M Fralick J Odutayo A Fralick M . Sodium-glucose cotransporter-2 inhibitors and risk of diabetic ketoacidosis among adults with type 2 diabetes: a systematic review and meta-analysis. Can J Diabetes. (2022) 46:e2.10–5. doi: 10.1016/j.jcjd.2021.04.006 34116926

[9] Handelsman Y Henry RR Bloomgarden ZT Dagogo-Jack S DeFronzo RA Einhorn D . American Association of Clinical Endocrinologists and American College of Endocrinology position statement on the association of SGLT-2 inhibitors and diabetic ketoacidosis. Endocrine Pract. (2016) 22:753–62. doi: 10.4158/EP161292.PS 27082665

[10] He Z Lam K Zhao W Yang S Li Y Mo J . SGLT-2 inhibitors and euglycemic diabetic ketoacidosis/diabetic ketoacidosis in FAERS: a pharmacovigilance assessment. Acta Diabetol. (2023) 60:401–11. doi: 10.1007/s00592-022-02015-6 36576563

[11] Thaibah HA Banji OJF Banji D Alshammari TM . Diabetic ketoacidosis and the use of new hypoglycemic groups: Real-world evidence utilizing the Food and Drug Administration Adverse Event Reporting System. Pharm (Basel). (2025) 18 (2):214. doi: 10.3390/ph18020214 40006028 PMC11858861

[12] Sakamoto T Miyamoto H Hashizume J Akamatsu H Akagi T Kodama Y . Differences in the adverse event profiles of sodium-glucose cotransporter 2 inhibitors used in patients with diabetes mellitus and heart failure: An analysis using the Japanese Adverse Drug Event Report Database. Clin Drug Investig. (2024) 44:761–71. doi: 10.1007/s40261-024-01394-8 39402407

[13] Du P Chen X Xu Y Zhou Y Gao H . Drug‐associated ketoacidosis: A comprehensive disproportionality analysis based on the FAERS database. Diabetes Obes Metab. (2026) 28:5893–906. doi: 10.1111/dom.70790 42010884

[14] Fusaroli M Salvo F Begaud B AlShammari TM Bate A Battini V . The reporting of a disproportionality analysis for drug safety signal detection using individual case safety reports in pharmacovigilance (READUS-PV): Development and statement. Drug Saf. (2024) 47:575–84. doi: 10.1007/s40264-024-01421-9 38713346 PMC11116242

[15] von Elm E Altman DG Egger M Pocock SJ Gøtzsche PC Vandenbroucke JP . The strengthening the reporting of observational studies in epidemiology (STROBE) statement: Guidelines for reporting observational studies. Lancet. (2007) 370:1453–7. doi: 10.1016/s0140-6736(07)61602-x 18064739

[16] Sakaeda T Tamon A Kadoyama K Okuno Y . Data mining of the public version of the FDA Adverse Event Reporting System. Int J Med Sci. (2013) 10:796–803. doi: 10.7150/ijms.6048 23794943 PMC3689877

[17] Tsuchiya M Obara T Sakai T Nomura K Takamura C Mano N . Quality evaluation of the Japanese Adverse Drug Event Report database (JADER). Pharmacoepidemiol Drug Saf. (2020) 29:173–81. doi: 10.1002/pds.4944 31823506

[18] Brown EG Wood L Wood S . The medical dictionary for regulatory activities (MedDRA). Drug Saf. (1999) 20:109–17. doi: 10.2165/00002018-199920020-00002 10082069

[19] van Puijenbroek EP Bate A Leufkens HG Lindquist M Orre R Egberts AC . A comparison of measures of disproportionality for signal detection in spontaneous reporting systems for adverse drug reactions. Pharmacoepidemiol Drug Saf. (2002) 11:3–10. doi: 10.1002/pds.668 11998548

[20] Evans SJ Waller PC Davis S . Use of proportional reporting ratios (PRRs) for signal generation from spontaneous adverse drug reaction reports. Pharmacoepidemiol Drug Saf. (2001) 10:483–6. doi: 10.1002/pds.677 11828828

[21] W D . Bayesian data mining in large frequency tables, with an application to the FDA spontaneous reporting system. Am Stat. (1999) 53:177–90.

[22] Bate A Evans SJ . Quantitative signal detection using spontaneous ADR reporting. Pharmacoepidemiol Drug Saf. (2009) 18:427–36. doi: 10.1002/pds.1742 19358225

[23] Harpaz R DuMouchel W LePendu P Bauer-Mehren A Ryan P Shah NH . Performance of pharmacovigilance signal-detection algorithms for the FDA adverse event reporting system. Clin Pharmacol Ther. (2013) 93:539–46. doi: 10.1038/clpt.2013.24 23571771 PMC3857139

[24] Danne T Garg S Peters AL Buse JB Mathieu C Pettus JH . International consensus on risk management of diabetic ketoacidosis in patients with type 1 diabetes treated with sodium-glucose cotransporter (SGLT) inhibitors. Diabetes Care. (2019) 42:1147–54. doi: 10.2337/dc18-2316 30728224 PMC6973545

[25] Taylor SI Blau JE Rother KI . SGLT2 inhibitors may predispose to ketoacidosis. J Clin Endocrinol Metab. (2015) 100:2849–52. doi: 10.1210/jc.2015-1884 26086329 PMC4525004

[26] Tomar VB Cetin TE Demirezen A Akcay OF Derici U Guz G . Predictors of asymptomatic ketonemia in outpatients with diabetic kidney disease receiving SGLT-2 inhibitors: a cross-sectional study. Diabetol Metab Syndr. (2026) 18 (1):174. doi: 10.1186/s13098-026-02200-5 42271508 PMC13479832

[27] Yeung SL Wang M Lou M Ng TMH . Sodium-glucose cotransporter-2 inhibitors and ketoacidosis in heart failure: Analysis of US Adverse Event Reporting System (FAERS). Eur Heart J Open. (2025) 5:oeaf056. doi: 10.1093/ehjopen/oeaf056 40438086 PMC12117328

[28] Khouri C Hlavaty A Fusaroli M Joshi N Salvo F Bate A . The rising misuse of pharmacovigilance reporting systems: A threat to evidence-based medicine. Clin Pharmacol Ther. (2026) 119:318–21. doi: 10.1002/cpt.70180 41422480

