# Supplementary material for: Disproportionate reporting of euglycaemic and diabetic ketoacidosis with SGLT-2 inhibitors across expanding cardiorenal indications: a cross-database study of FAERS and JADER
Source: Front Endocrinol (Lausanne). 2026 Sep 3;17:1957734. doi: 10.3389/fendo.2026.1957734 (PMC13538859; doi:10.3389/fendo.2026.1957734)
Supplement: Supplementary file 1 [file DataSheet1.docx]

**Supporting Information**

*For: “Disproportionate reporting of euglycaemic and diabetic ketoacidosis with SGLT-2 inhibitors across expanding cardiorenal indications: a cross-database study of FAERS and JADER.” Each item is cited in the main text by its table or figure number.*

**Contents**:

Table S1 — MedDRA preferred terms for outcome clusters;

Table S2 — full 2×2 cell counts;

Table S3 — active-comparator analysis (vs DPP-4 inhibitors);

Table S4 — calendar-period analysis;

Figure S1 — report-selection flow diagram;

Table S5 — sensitivity analyses; Figure S2 — proposed mechanism;

Table S6 — calculation formulas and decision thresholds;

Table S7 — time-to-onset completeness;

Table S8 — cross-database comparison;

Table S9 — outcome-definition sensitivity (wide vs narrow DKA);

READUS-PV reporting checklist;

STROBE reporting checklist.

**MedDRA preferred terms used for outcome definitions**

Table S1. Preferred terms (PTs) composing each outcome cluster (MedDRA Preferred Terms as coded in the source data, stable across the MedDRA versions spanning the study window (v23.0–v28.1); MedDRA/J, whose English Preferred Terms are identical, used for JADER).

| **Outcome** | **MedDRA preferred terms (English)** |
| --- | --- |
| DKA cluster (included) | Diabetic ketoacidosis; Euglycaemic diabetic ketoacidosis; Ketoacidosis; Ketosis; Metabolic acidosis. JADER (Japanese MedDRA): 糖尿病性ケトアシドーシス; 正常血糖糖尿病性ケトアシドーシス; ケトアシドーシス; 糖尿病性ケトーシス; ケトーシス; ケトーシス傾向糖尿病; 代謝性アシドーシス; 糖尿病性ケトアシドーシス性高血糖昏睡 |
| Excluded from DKA cluster (non-specific acidoses and isolated laboratory values) | Alcoholic ketoacidosis; Starvation ketoacidosis; Lactic acidosis; Renal tubular acidosis; Respiratory acidosis; Foetal/neonatal acidosis; Hyperchloraemic acidosis; isolated blood/urine ketone-body laboratory values (present/absent/increased/decreased). JADER equivalents excluded: 乳酸アシドーシス; アルコール性ケトアシドーシス; 飢餓性ケトアシドーシス; 腎尿細管性アシドーシス; 呼吸性アシドーシス; 胎児アシドーシス; 高クロール血性アシドーシス; 新生児の遅発代謝性アシドーシス; 尿中/血中ケトン体（陽性・増加）; bare アシドーシス |
| euDKA | Euglycaemic diabetic ketoacidosis (exact term). JADER: 正常血糖糖尿病性ケトアシドーシス |
| Negative control 1 — fracture | Fracture; Bone fracture. JADER: 骨折 and site-specific fracture terms (pathological/traumatic/infection-qualified fracture terms excluded) |
| Negative control 2 — road traffic accident | Road traffic accident. JADER: 交通事故 |

*euDKA was analysed using the specific euglycaemic-ketoacidosis term to avoid overlap with the general DKA cluster.*

**Full 2×2 cell counts for all analyses**

Table S2. Contingency cell counts (a = exposure & event; b = exposure & non-event; c = non-exposure & event; d = non-exposure & non-event) underlying every reported estimate.

| **Analysis** | **a** | **b** | **c** | **d** | **ROR (95% CI)** |
| --- | --- | --- | --- | --- | --- |
| FAERS — overall |  |  |  |  |  |
| DKA | 8779 | 58686 | 19999 | 9012751 | 67.42 (65.66–69.22) |
| euDKA | 2783 | 64682 | 461 | 9032289 | 843 (763.65–930.6) |
| FRACTURE | 45 | 67420 | 9067 | 9023683 | 0.66 (0.5–0.89) |
| RTA | 89 | 67376 | 15844 | 9016906 | 0.75 (0.61–0.93) |
| FAERS — by indication (DKA) |  |  |  |  |  |
| T2DM | 5733 | 18680 | 3977 | 200337 | 15.46 (14.81–16.14) |
| HF | 360 | 4811 | 194 | 36756 | 14.18 (11.88–16.92) |
| CKD | 95 | 1595 | 73 | 19490 | 15.9 (11.67–21.67) |
| T1DM | 252 | 180 | 940 | 11903 | 17.73 (14.48–21.71) |
| FAERS — vs DPP-4i (DKA) |  |  |  |  |  |
| SGLT-2i vs DPP-4i | 8779 | 58686 | 780 | 18448 | 3.54 (3.28–3.81) |
| JADER — overall |  |  |  |  |  |
| DKA | 1535 | 5973 | 2340 | 1022179 | 112.26 (104.75–120.31) |
| euDKA | 589 | 6919 | 53 | 1024466 | 1645.48 (1241.06–2181.7) |
| FRACTURE | 85 | 7423 | 10312 | 1014207 | 1.13 (0.91–1.4) |
| RTA | 5 | 7503 | 683 | 1023836 | 1 (0.41–2.41) |
| JADER — by indication (DKA) |  |  |  |  |  |
| T2DM | 875 | 3524 | 218 | 15147 | 17.25 (14.81–20.1) |
| HF | 103 | 703 | 22 | 13995 | 93.2 (58.45–148.61) |
| CKD | 25 | 381 | 14 | 4744 | 22.23 (11.46–43.13) |
| T1DM | 253 | 48 | 111 | 1583 | 75.17 (52.25–108.14) |
| JADER — vs DPP-4i (DKA) |  |  |  |  |  |
| SGLT-2i vs DPP-4i | 1535 | 5973 | 241 | 11359 | 12.11 (10.54–13.92) |

*a/b/c/d are report counts. ROR with 95% CI reproduced for cross-checking. Indication strata are defined at the report level (a report may contribute to more than one stratum). The active-comparator rows use DPP-4 inhibitor reports as the reference (cell d = DPP-4i non-DKA reports).*

**Active-comparator analysis (SGLT-2i vs DPP-4 inhibitors), detailed**

Table S3. Disproportionality of DKA for SGLT-2 inhibitors relative to DPP-4 inhibitors, all methods.

| **Database** | **a** | **b** | **c** | **d** | **ROR (95% CI)** | **PRR** | **IC025** |
| --- | --- | --- | --- | --- | --- | --- | --- |
| FAERS | 8779 | 58686 | 780 | 18448 | 3.54 (3.28–3.81) | 3.21 | 0.2 |
| JADER | 1535 | 5973 | 241 | 11359 | 12.11 (10.54–13.92) | 9.84 | 1.04 |

*Reference group = DPP-4 inhibitor reports. ROR > 1 indicates disproportionately more DKA reporting with SGLT-2 inhibitors than with DPP-4 inhibitors, i.e. the signal is not explained by the shared glucose-lowering/diabetes reporting context.*

**Calendar-period analysis (2020–2022 vs 2023–2026)**

Within-indication DKA disproportionality (SGLT-2 inhibitor versus all other drugs) was computed separately for reports received in 2020–2022 and 2023–2026, using the tightened DKA definition. The signal remained significant in every stratum in both periods, with consistent direction. Magnitudes were somewhat higher in the earlier period (overall ROR 123.7 vs 41.2), as expected when an exposure is newer and reporting is more event-driven, but the persistence of a strong signal in the most recent period argues against post-approval reporting growth (the Weber effect) as the explanation.

*Table S4. Calendar-period DKA disproportionality (SGLT-2 inhibitor vs all other drugs, tightened DKA definition), by stratum. ROR (95% CI).*

| **Stratum** | **2020–2022 ROR (95% CI)** | **2023–2026 ROR (95% CI)** |
| --- | --- | --- |
| Overall | 123.7 (119.2–128.4) | 41.2 (39.7–42.8) |
| T2DM | 16.9 (15.9–17.9) | 14.0 (13.2–14.9) |
| HF | 23.7 (18.1–31.1) | 8.4 (6.6–10.7) |
| CKD | 16.1 (8.7–30.0) | 16.7 (11.7–24.0) |
| T1DM | 24.5 (18.8–31.9) | 8.9 (6.2–12.6) |

**Report selection flow diagram**

Figure S1. Flow of report selection for FAERS and JADER, from all reports in the study window to the SGLT-2 inhibitor exposure set and each outcome, with de-duplication and exclusion counts at each step.

**Table S5.** Sensitivity analyses of the DKA disproportionality signal

| **Database / stratum** | **Main analysis** | **S1: primary-suspect only** | **S2: precipitants excluded** |
| --- | --- | --- | --- |
| FAERS |  |  |  |
| Overall | 67.4 (65.7–69.2) | 57.2 (55.6–58.8) | 80.1 (77.7–82.6) |
| T2DM | 15.5 (14.8–16.1) | 10.5 (10.0–10.9) | 17.1 (16.3–18.0) |
| HF | 14.2 (11.9–16.9) | 10.1 (8.5–12.0) | 20.1 (15.7–25.7) |
| CKD | 15.9 (11.7–21.7) | 11.3 (8.3–15.4) | 14.8 (10.0–21.9) |
| off-label T1DM | 17.7 (14.5–21.7) | 19.5 (15.8–24.1) | 15.4 (12.6–18.9) |
| JADER |  |  |  |
| Overall | 112.3 (104.8–120.3) | 112.3 (104.8–120.3) | 111.9 (104.0–120.4) |
| T2DM | 17.2 (14.8–20.1) | 17.2 (14.8–20.1) | 17.0 (14.4–20.1) |
| HF | 93.2 (58.5–148.6) | 93.2 (58.5–148.6) | 139.4 (75.8–256.2) |
| CKD | 22.2 (11.5–43.1) | 22.2 (11.5–43.1) | 24.5 (10.9–54.9) |
| off-label T1DM | 75.2 (52.2–108.1) | 75.2 (52.2–108.1) | 64.0 (45.2–90.8) |

*DKA, diabetic ketoacidosis; ROR, reporting odds ratio; CI, confidence interval. Values are ROR (95% CI). S1 restricts to reports in which an SGLT-2 inhibitor was the primary suspect drug. S2 excludes reports listing acute precipitants of ketosis (infection, fasting, dehydration, vomiting, surgery, acute decompensation, myocardial infarction). All estimates met four-method consensus. In JADER, SGLT-2 inhibitor exposure was defined as suspect drug in the main analysis, so S1 is identical to the main analysis by construction.*

**Figure S2.** Proposed mechanism of SGLT-2 inhibitor–associated euglycaemic ketoacidosis. SGLT-2 (SLC5A2) inhibition produces persistent glucosuria and a low insulin/high glucagon state, driving lipolysis and hepatic ketogenesis; because glucose continues to be excreted, ketoacidosis can develop with near-normal blood glucose (euDKA). Acute precipitants (infection, fasting, surgery, dehydration) act as amplifiers rather than the principal driver

**Table S6**. Calculation formulas and decision thresholds for the four disproportionality methods

| **Method** | **Calculation** | **Signal threshold** |
| --- | --- | --- |
| ROR | (a/c)/(b/d); SE(lnROR) = √(1/a+1/b+1/c+1/d); 95% CI = exp(lnROR ± 1.96×SE) | a ≥ 3 and lower 95% CI > 1 |
| PRR | [a/(a+b)] / [c/(c+d)]; χ² with Yates continuity correction: (\|a − E11\| − 0.5)² / E11, where E11 = (a+b)(a+c)/N | a ≥ 3, PRR ≥ 2 and χ² ≥ 4 |
| IC (BCPNN) | log₂[aN / ((a+b)(a+c))]; IC025 = IC − 1.96√V(IC) | IC025 > 0 |
| EBGM (MGPS) | Empirical Bayes geometric mean with gamma–Poisson shrinkage (openEBGM package); EB05 = lower 5% bound | EB05 > 2 |

*a, reports with both the drug and the event; b, reports with the drug but not the event; c, reports with the event but not the drug; d, neither; N = a+b+c+d. A signal was declared only when all four criteria were met simultaneously. Analyses were performed in R 4.4.2.*

**Table S7**. Completeness of time-to-onset data: characteristics of reports with versus without a usable TTO

| **Characteristic** | **Without usable TTO (n=6,699)** | **With usable TTO (n=2,080)** | **Test** | **p** |
| --- | --- | --- | --- | --- |
| Reporter occupation |  |  | Pearson’s χ² | <0.001 |
| Physician (MD) | 50.1% | 45.3% |  |  |
| Pharmacist (PH) | 13.0% | 26.0% |  |  |
| Other health professional (HP) | 23.9% | 16.1% |  |  |
| Consumer (CN) | 7.1% | 6.4% |  |  |
| Not recorded | 5.8% | 6.3% |  |  |
| Sex |  |  | Pearson’s χ² | <0.001 |
| Female | 40.2% | 42.7% |  |  |
| Male | 46.0% | 55.4% |  |  |
| Not recorded | 13.8% | 1.9% |  |  |
| Age, years (median) | 61 | 60 | Wilcoxon rank-sum | 0.025 |

*TTO, time to onset. Denominator is the 8,779 FAERS reports in which an SGLT-2 inhibitor was a suspect drug and a diabetic ketoacidosis preferred term was recorded. A usable TTO required complete (YYYYMMDD) therapy-start and event dates and an interval of 1–3650 days. The difference in age reached statistical significance but is negligible in magnitude. Reporter country and report seriousness were not available in the analytic file. Reporter occupation and sex were compared by Pearson’s chi-squared test, and age by the Wilcoxon rank-sum (Mann–Whitney U) test.*

**Table S8**. Cross-database comparison of reporting odds ratios (FAERS versus JADER)

| **Comparison** | **FAERS ROR** | **JADER ROR** | **JADER/FAERS ratio** |
| --- | --- | --- | --- |
| Overall DKA | 67.42 | 112.3 | 1.67 |
| Overall euDKA | 843 | 1645 | 1.95 |
| Type 2 diabetes | 15.46 | 17.3 | 1.12 |
| Heart failure | 14.18 | 93.2 | 6.57 |
| Chronic kidney disease | 15.90 | 22.2 | 1.40 |
| Off-label type 1 diabetes | 17.73 | 75.2 | 4.24 |
| Versus DPP-4 inhibitors | 3.54 | 12.1 | 3.42 |
| Negative control: fracture | 0.66 | 1.13 | 1.71 |
| Negative control: road traffic accident | 0.75 | 1.00 | 1.33 |

*ROR, reporting odds ratio. Median ratio across the nine paired comparisons 1.95 (range 1.12–6.57); Spearman ρ=0.88, p=0.002. Restricted to the seven positive signals, median ratio 1.95 (interquartile range 1.53–3.83) and ρ=0.79, p=0.036. The two largest ratios occur in the strata with the fewest reports.*

**Table S9**. DKA-cluster disproportionality in FAERS and JADER under the primary (narrow, five preferred terms) versus a broadened (wide) outcome definition. The wide definition returns the acidosis and ketone-related preferred terms excluded from the primary cluster (Table S1); SGLT-2 inhibitors versus all other drugs. euDKA is defined by a single preferred term and is unaffected.

| **Database** | **Definition** | **a** | **b** | **c** | **d** | **ROR (95% CI)** | **PRR** | **IC025** | **Signal** |
| --- | --- | --- | --- | --- | --- | --- | --- | --- | --- |
| FAERS | Narrow (5 PTs) | 8779 | 58 686 | 19 999 | 9 012 751 | 67.4 (65.7–69.2) | 58.8 | 5.33 | Met (all four) |
| FAERS | Wide (+excluded) | 9824 | 57 641 | 37 832 | 8 994 918 | 40.5 (39.6–41.5) | 34.8 | 4.76 | Met (ROR/PRR/IC) |
| JADER | Narrow (5 PTs) | 1535 | 5973 | 2340 | 1 022 179 | 112.3 (104.8–120.3) | 89.5 | 5.68 | Met (all four) |
| JADER | Wide (+excluded) | 1659 | 5849 | 5082 | 1 019 437 | 56.9 (53.5–60.5) | 44.5 | 5.00 | Met (ROR/PRR/IC) |

*a–d, contingency cell counts. All three closed-form measures met their signal criteria under both definitions (ROR lower bound >1; PRR ≥2 with Yates-corrected χ²≥4; IC025 >0). The EBGM/EB05 (openEBGM) is fitted on the whole database and was computed for the primary analysis (main text, Table 1); this outcome-definition sensitivity analysis reports the three closed-form measures. The lower ROR under the wide definition reflects the inclusion of non-specific acidoses (e.g. alcoholic, starvation, lactic) that are not preferentially reported with SGLT-2 inhibitors.*

**READUS-PV reporting checklist (completed)**

*Completed against the 14 main items (with sub-items) and the abstract items of the READUS-PV statement (Fusaroli et al., Drug Saf 2024;47:575–584 and 585–599). “Location” gives the section, table or figure where each item is addressed. “Not applicable” items are justified in the adjacent cell.*

| **Item** | **READUS-PV recommendation (condensed)** | **Location / how addressed in this manuscript** |
| --- | --- | --- |
| **Title** | | |
| **1a** | Identify the study as a 'disproportionality analysis'; specify data type (ICSRs) and database(s). | Title (“Disproportionate reporting … a cross-database study of FAERS and JADER”) names both databases; ‘Disproportionality analysis’ is a Keyword; ICSR data type stated in §2.1. |
| **1b** | Report the adverse event(s) and/or drug(s) under study. | Title names the events (euglycaemic and diabetic ketoacidosis), the drug class (SGLT-2 inhibitors) and the population (expanding cardiorenal indications). |
| **Introduction** | | |
| **2a** | Describe the drug(s), the adverse event(s) and their frequency, and existing knowledge; identify the gap. | §1 ¶1 (SGLT-2i indications/expansion); ¶2 (nature of DKA/euDKA, near-normal glucose, precipitants, frequency ‘well under one per thousand’); ¶3 (three explicit knowledge gaps). |
| **2b** | Specify the rationale for performing the analysis (e.g. prespecified hypothesis). | §1 ¶3–¶4 and §2 opening: hypothesis-generating test of whether the signal is maintained, diluted or amplified as prescribing expands. |
| **2c** | Explain why ICSR databases and disproportionality analysis are suitable, and the added value versus prior work. | §1 ¶3 (spontaneous databases ‘well suited’); §1 ¶4 and §4.3 delimit He, Sakamoto, Yeung and Du and state the added value (whole-background reference, JADER replication, indication strata, negative controls). |
| **3** | State specific objectives: adverse event(s), drug(s), reference group and any prespecified hypothesis. | §1 final ¶: indication-stratified DA of SGLT-2i–associated DKA/euDKA; reference = all other drugs, with DPP-4 inhibitors as active comparator. |
| **Methods** | | |
| **4a** | Identify the study and the type of data used. | §2.1: ‘disproportionality analysis of individual case safety reports (ICSRs)’. |
| **4b** | Outline the whole design, including primary and sensitivity analyses and any case-by-case / literature review. | §2 opening + §2.4: overall, indication-stratified, DPP-4i comparator, two negative controls, time-to-onset, and suspect-only / precipitant-excluded / calendar-period sensitivity analyses. No case-by-case analysis (see 7d/10). |
| **5a** | Database name, custodian, coverage; product type; coding thesaurus and version; size. | §2.1 (FAERS [US FDA], 2020Q1–2026Q1, URL; JADER [PMDA], cumulative May 2026, URL; sizes in §3.1); MedDRA coding, §2.3 + Table S1. MedDRA Preferred Terms used as coded in the source data (stable across v23.0–v28.1 over the study window); MedDRA/J for JADER. Stated in Table S1. |
| **5b** | Extraction dates and all preprocessing choices (deduplication, missing data, exclusions), justified. | §2.1 (deduplication: latest CASEVERSION/PRIMARYID per CASEID; deleted-case exclusion); §2.4 (time-to-onset exclusions: missing / non-positive / >10 years). |
| **6a** | Describe the study population and any restriction. | §2.2: SGLT-2 inhibitors as a suspect drug (incl. fixed-dose combinations); report-level indication strata (T2DM, HF, CKD, off-label T1DM). |
| **6b** | Describe the nature and meaning of key variables and the handling of missing values. | §2.3 (outcome clusters); §2.4 and §4.4 + Table S7 (time-to-onset completeness and missing-date handling). |
| **6c** | Specify and justify drug/event groupings, drug roles, and active-ingredient / trade-name choices. | §2.2 (suspect roles PS/SS; full agent list in the Supporting Information) + §2.3 (five-PT DKA cluster; euDKA preferred term; negative-control PTs; Table S1). |
| **6d** | Describe any additional data source and how it interacts with ICSRs. | Not applicable — analysis is confined to two ICSR databases; no external dataset was linked. |
| **7a** | Present any descriptive analysis (variables, tests, thresholds). | §2.4 (exposure-background and case-attribution proportions) → §3.2 / Table 2. |
| **7b** | Disproportionality measure(s), dimensionality, units, comparator group and SDR thresholds; rationale. | §2.4: ROR, PRR, IC (IC025) and MGPS (EBGM/EB05); four-method consensus; units = reports; comparators = all other drugs and DPP-4 inhibitors; formulas/thresholds in Table S6. |
| **7c** | Describe sensitivity analyses and tools to control confounding (restriction, subgroup, stratification). | §2.4 + §3.6–3.7: primary-suspect-only and precipitant-excluded analyses (Table S5); calendar-period split 2020–22 vs 2023–26 (Table S4). |
| **7d** | Variables and methods for case-by-case causality assessment, if performed. | Not applicable — not performed because case narratives are not routinely accessible in FAERS or JADER (§4.4). |
| **7e** | Statistical methods used for other data sources. | Not applicable — no other data sources. Software: R 4.4.2 with openEBGM (§2.4). |
| **Results** | | |
| **8a** | Number of ICSRs included/excluded at each stage, with reasons. | §3.1 (≈9.1 M FAERS / 1,032,027 JADER analysed; SGLT-2i suspect in 67,465 / 7,508); report-selection flow in Figure S1. |
| **8b** | Key demographic/clinical characteristics of cases, versus a reference group where possible. | Descriptive results in §3.2 (Table 2) and time-to-onset-subset completeness (reporter occupation, sex missingness, age) in §4.4 / Table S7. A full case-level demographic table is not presented, as cases span two databases and overlapping strata. |
| **9** | Present all results incl. confidence intervals and sensitivity analyses; give contingency-table figures and expected counts. | §3.1–3.8 (ROR/PRR/IC/EBGM with 95% CIs; Tables 1–3; Figs 1–3); sensitivity in Table S5; full 2×2 cell counts (a,b,c,d) in Table S2 (expected counts derivable). |
| **10** | Present case-by-case results and causality assessment, if applicable. | Not applicable — case-by-case analysis not performed (narratives unavailable; §4.4). |
| **Discussion** | | |
| **11** | Discuss key results vs objectives; distinguish expected reactions from emerging signals; context, plausibility, negative controls. | §4 opening (three key findings); §4.1 (mechanism); §4.3 (comparison with prior work); negative-control nullity in §3.1 / §4. |
| **12a** | Discuss external validity to the general population. | §4 (recorded indication reflects context of use, not individual metabolic state; cannot establish risk specifically in non-diabetic patients) + §4.4. |
| **12b** | Discuss potential relevance in clinical practice. | §4.2: ketone-based assessment soon after initiation, across the expanding cardiorenal indications. |
| **12c** | Propose further study designs, if applicable. | §4.4: ‘the natural next step is a denominator-based, real-world cohort …’. |
| **13** | General + specific limitations; state DA cannot prove causation or measure incidence; confounding/reporting bias and mitigation. | §4.4: extensive; explicit that estimates quantify disproportionality of reporting and ‘cannot provide incidence or causal estimates’; stratum overlap, time-to-onset subset bias, cross-database non-comparability all stated. |
| **Declarations** | | |
| **14a** | Source of funding/sponsorship and the role of funders. | ‘Funding’ (NHC grant WKZX2024CX501224) and ‘Acknowledgements’ (Tianjin Key Medical Discipline TJYXZDXK-3-007B). Funding states that the funders had no role in study design, data collection, analysis, interpretation, or manuscript preparation. |
| **14b** | Commercial/intellectual conflicts of interest. | ‘Competing interests’ — none declared. |
| **14c** | Institutional approval needed or granted. | ‘Ethics approval’ — exempt (publicly available, fully de-identified data). |
| **14d** | Data/code availability, software version, and protocol registration. | ‘Data availability’ (FAERS/JADER public; code on request); software R 4.4.2 (§2.4). analysis plan pre-specified before data extraction (Methods §2); study protocol not publicly registered, stated in Data availability. |
| **Abstract (READUS-PV for Abstracts)** | | |
| **A1** | Identify as a disproportionality analysis and name the database(s); keywords covering population/drug/event. | Abstract and Keywords name SGLT-2 inhibitors, euDKA/DKA and FAERS/JADER; ‘Disproportionality analysis’ among Keywords. |
| **A2** | State the objective(s). | Abstract ‘Aims’: whether the ketoacidosis/euDKA signal is maintained as indications expand and reproduces across systems. |
| **A3** | Summarise methods (databases, measures, thresholds). | Abstract ‘Methods’: FAERS + JADER; four-method consensus (ROR/PRR/IC/EBGM); strata; DPP-4i comparator; negative controls; time-to-onset. |
| **A4** | Report results for all main outcomes with CIs, and state DA cannot establish incidence or causality. | Abstract ‘Results/Conclusions’ give RORs with 95% CIs and null negative controls. abstract Conclusions state these are hypothesis-generating signals that cannot establish incidence, relative risk, or causality. |

**STROBE reporting checklist (completed)**

*Completed against the 22 items of the STROBE statement for observational (cross-sectional) studies (von Elm et al., Lancet 2007;370:1453-1457). Several items overlap the READUS-PV checklist above; both are provided because DOM lists STROBE for observational studies. “Location” gives the section, table or figure where each item is addressed.*

| **Item** | **STROBE recommendation (condensed)** | **Location / how addressed in this manuscript** |
| --- | --- | --- |
| **Title and abstract** | | |
| **1** | (a) Indicate the study design in the title or abstract; (b) give an informative, balanced summary. | (a) Title ‘cross-database study’; ‘disproportionality analysis’ among Keywords. (b) Structured abstract (Aims / Materials and Methods / Results / Conclusions) with the hypothesis-generating caveat. |
| **Introduction** | | |
| **2** | Background/rationale. | §1 ¶1–¶3 (SGLT-2i indication expansion; DKA/euDKA nature and frequency; three knowledge gaps). |
| **3** | State specific objectives, including any prespecified hypotheses. | §1 final ¶: indication-stratified DA of SGLT-2i–associated DKA/euDKA, with JADER replication; hypothesis that the signal is maintained as indications expand. |
| **Methods** | | |
| **4** | Present key elements of study design early. | §2.1 (disproportionality analysis of ICSRs); §2.4 (design outline: overall, stratified, comparator, negative controls, sensitivity). |
| **5** | Setting, locations, relevant dates. | §2.1: FAERS (US FDA, 2020Q1–2026Q1) and JADER (PMDA, cumulative to May 2026); extraction and de-duplication described. |
| **6** | Eligibility criteria; sources and methods of selection. | §2.2: reports with SGLT-2 inhibitors as a suspect drug; report-level indication strata (T2DM, HF, CKD, off-label T1DM); case/non-case framework in §2.4. |
| **7** | Clearly define outcomes, exposures, predictors, potential confounders and effect modifiers. | §2.3 (five-PT DKA cluster; euDKA term; two negative controls; Table S1); §2.2 (exposure and indication strata); DPP-4i active comparator (§2.4). |
| **8** | For each variable, give sources of data and details of measurement. | §2.1 (database fields, de-duplication); §2.3 (MedDRA Preferred-Term coding); §2.4 (time-to-onset from therapy-start and event dates). |
| **9** | Describe any efforts to address potential sources of bias. | §2.4 and §4.4: two pre-specified negative controls, active comparator, primary-suspect-only and precipitant-excluded sensitivity analyses, calendar-period split (notoriety/Weber); reporting-bias limitations discussed. |
| **10** | Explain how the study size was arrived at. | §3.1: entire FAERS (≈9.1 M) and JADER (1,032,027) reports in the window; SGLT-2i suspect in 67,465 / 7,508 (no sampling). |
| **11** | Explain how quantitative variables were handled; groupings and why. | §2.2–2.3 (PT and indication groupings, with rationale); §2.4 (four disproportionality measures; time-to-onset as continuous, Weibull). |
| **12** | Statistical methods: (a) main; (b) subgroups/interactions; (c) missing data; (d) sampling; (e) sensitivity analyses. | §2.4 + Table S6: (a) four-method consensus (ROR/PRR/IC/EBGM); (b) indication strata and comparator; (c) time-to-onset missing-date handling (§2.4, Table S7); (d) whole-database, no sampling; (e) suspect-only, precipitant-excluded, calendar-period. |
| **Results** | | |
| **13** | (a) Numbers at each stage; (b) reasons for non-participation; (c) flow diagram. | §3.1 (reports analysed and SGLT-2i-exposed at each stage; exclusions); flow diagram Figure S1. |
| **14** | (a) Characteristics of participants; (b) missing data per variable. | §3.2 (descriptive proportions); Table S7 (time-to-onset-subset completeness and missingness by reporter/sex/age). |
| **15** | Report numbers of outcome events or summary measures. | §3.1–3.8: report counts underlying each estimate; full 2×2 cell counts (a,b,c,d) in Table S2. |
| **16** | (a) Estimates with confidence intervals; (b) category boundaries; (c) translate to absolute risk if relevant. | §3.1–3.8: ROR/PRR/IC/EBGM with 95% CIs (Tables 1–3; Figs 1–3). (c) Absolute risk not estimated — stated as a limitation (§4.4). |
| **17** | Other analyses (subgroups, interactions, sensitivity). | §3.6–3.7 (sensitivity; calendar-period); §3.8 (cross-database concordance, Figure 3, Table S8). |
| **Discussion** | | |
| **18** | Summarise key results with reference to objectives. | §4 opening: three key findings tied to the objectives. |
| **19** | Limitations, direction and magnitude of potential bias. | §4.4: reporting/indication bias, stratum overlap, time-to-onset subset bias, cross-database non-comparability; explicit that DA cannot establish incidence or causality. |
| **20** | Cautious overall interpretation considering objectives, limitations, other evidence. | §4.1 (mechanism), §4.2 (clinical relevance), §4.3 (comparison with prior work). |
| **21** | Generalisability (external validity). | §4 and §4.4: recorded indication reflects context of use, not individual metabolic state; cannot generalise to incidence in non-diabetic patients. |
| **Other information** | | |
| **22** | Source of funding and the role of funders. | Funding (NHC grant WKZX2024CX501224); funders had no role in the study. |
